# Combination treatment with doxorubicin and gamitrinib synergistically augments anticancer activity through enhanced activation of Bim

**DOI:** 10.1186/1471-2407-14-431

**Published:** 2014-06-13

**Authors:** Hye-Kyung Park, Ji-Eun Lee, Jaehwa Lim, Da-Eun Jo, Soo-Ah Park, Pann-Ghill Suh, Byoung Heon Kang

**Affiliations:** 1 Department of Biological Sciences, School of Life Sciences, UNIST, 50 UNIST St., Ulsan 689-798, South Korea

## Abstract

**Background:**

A common approach to cancer therapy in clinical practice is the combination of several drugs to boost the anticancer activity of available drugs while suppressing their unwanted side effects. In this regard, we examined the efficacy of combination treatment with the widely-used genotoxic drug doxorubicin and the mitochondriotoxic Hsp90 inhibitor gamitrinib to exploit disparate stress signaling pathways for cancer therapy.

**Methods:**

The cytotoxicity of the drugs as single agents or in combination against several cancer cell types was analyzed by MTT assay and the synergism of the drug combination was evaluated by calculating the combination index. To understand the molecular mechanism of the drug synergism, stress signaling pathways were analyzed after drug combination. Two xenograft models with breast and prostate cancer cells were used to evaluate anticancer activity of the drug combination *in vivo*. Cardiotoxicity was assessed by tissue histology and serum creatine phosphokinase concentration.

**Results:**

Gamitrinib sensitized various human cancer cells to doxorubicin treatment, and combination treatment with the two drugs synergistically increased apoptosis. The cytotoxicity of the drug combination involved activation and mitochondrial accumulation of the proapoptotic Bcl-2 family member Bim. Activation of Bim was associated with increased expression of the proapoptotic transcription factor C/EBP-homologous protein and enhanced activation of the stress kinase c-Jun N-terminal kinase. Combined drug treatment with doxorubicin and gamitrinib dramatically reduced *in vivo* tumor growth in prostate and breast xenograft models without increasing cardiotoxicity.

**Conclusions:**

The drug combination showed synergistic anticancer activities toward various cancer cells without aggravating the cardiotoxic side effects of doxorubicin, suggesting that the full therapeutic potential of doxorubicin can be unleashed through combination with gamitrinib.

## Background

Heat shock protein 90 (Hsp90) is an ATP-dependent molecular chaperone that controls folding of a wide range of protein substrates, or clients, many of which are involved in signal pathways crucial for tumorigenesis [1,2]. The primary cellular location of Hsp90 is the cytoplasm, but a pool of Hsp90 and its isoform, tumor necrosis factor receptor-associated protein 1 (TRAP1), has been reported in mitochondria [3,4]. The mitochondrial expression of Hsp90 and TRAP1 is often elevated in many cultured cancer cells and human cancer patients [3,5,6]. These proteins play important roles in multistep tumorigenic processes including the neoplastic metabolic shift to aerobic glycolysis [7-9] and inhibition of cell death [3].

A class of mitochondriotropic Hsp90 inhibitors, named gamitrinibs (GA mitochondrial matrix inhibitors), has been developed through combinatorial chemistry [10]. Gamitrinibs consist of geldanamycin, a competitive inhibitor of the ATPase pocket of Hsp90 and TRAP1, conjugated with tandem repeats of tetracyclic guanidinium or triphenylphosphonium for mitochondrial targeting [10,11]. Gamitrinibs not only trigger massive cell death in cultured cancer cells *in vitro* but also strongly suppress tumor growth in various xenograft and genetic mouse cancer models *in vivo*[10,12,13]. The gamitrinib-induced cytotoxicity is attributed to the reactivation of cyclophilin D (Cyp-D), an opener of the permeability transition pore (PTP) located in the mitochondrial inner membrane [3,14]. Because such opening of the PTP can be lethal, Cyp-D activation is often suppressed in cancer cells by interaction with mitochondrial Hsp90s, which increase resistance to various cellular stresses [3]. In addition, gamitrinibs have been shown to induce organelle-specific stress responses and dysregulation of bioenergetics in mitochondria of cancer cells, concomitantly compromising neoplastic growth [9,15-17].

Doxorubicin (DOX), an anthracycline antibiotic with the trade name Adriamycin, is one of the most effective anticancer drugs and has been widely used in various chemotherapeutic regimens to treat patients with cancer [18]. The antitumor activities of DOX are primarily attributed to DNA damage resulting from the inhibition of DNA topoisomerase II [18,19]. The clinical use of DOX, however, has been limited by the risk of cardiotoxicity, which is dependent on the cumulative dose/treatment schedule, typically refractory to common medications, and can be fatal [20-22].

Here, we examined whether a combination of two cytotoxic drugs with unrelated action mechanisms, DOX (genotoxic) and gamitrinib (mitochondriotoxic), would exhibit enhanced anticancer activity without aggravating unwanted side effects. This drug combination showed synergistically increased anticancer activities *in vitro* and *in vivo*, without augmenting cardiomyocyte toxicity. The underlying mechanism of action involved the activation of a proapoptotic Bcl-2 protein following the stimulation of CHOP and JNK pathways in cancer cells.

## Methods

### Chemicals and antibodies

Gamitrinib conjugated with triphenylphosphonium was prepared as described previously [10]. MitoTracker, JC-1, and tetramethylrhodamine methyl ester (TMRM) were purchased from Molecular Probes. All other chemicals were purchased from Sigma.

The following antibodies were used in this study: anti-JNK, anti-phospho-JNK (Thr183/Tyr185), anti-COX-IV, and anti-CHOP from Cell Signaling Technology; anti-cytochrome *c*, anti-Bim, and anti-PARP from Santa Cruz Biotechnology; anti-β-actin from MP Biomedicals; and anti-TRAP1, anti-Bax, anti-caspase-8, and anti-caspase-3 from BD Biosciences.

### Cells and cell culture

Human cancer cell types that originated from ovary (SK-OV3), prostate (22Rv1 and PC3), cervix (HeLa), breast (MDA-MB-231), liver (SK-HEP-1), brain (A172), kidney (ACHN), and lung (NCI-H460) were purchased from the Korean Cell Line Bank. Cells were cultured in DMEM or RPMI (GIBCO) medium containing 10% FBS (GIBCO) and 1% penicillin/streptomycin (GIBCO) at 37°C in a 10% CO_2_ humidified atmosphere.

### siRNA treatment

Small interfering RNAs (siRNAs) against JNK and CHOP were synthesized by Genolution Inc (Korea). siRNA sequences used in this study were as follows:

JNK1-#1, 5′-AAAGAATGTCCTACCTTCTCT-3′; JNK1-#2, 5′-AAGCCCAGTAATATAGTAGTA-3′; CHOP-#1, 5′-AGAACCAGCAGAGGTCACAA-3′; CHOP-#2, 5′-AAGAGAATGAACGGCTCAAGC-3′; Bim-#1, 5′-GCAACCTTCTGATGTAAGT-3′; Bim-#2, 5′-GACCGAGAAGGTAGACAATT-3′ and control, 5′-ACUCUAUCUGCACGCUGAC-3′. Cells were cultured on 6-well plates to 50–75% confluence, transfected with 40 nM siRNA mixed with G-Fectin (Genolution) for 48 hours, and then analyzed or treated with drugs.

### Analysis of cell viability and apoptosis induction

Cell viability was determined using 3(4,5-dimethyl-thyzoyl-2-yl)2,5 diphenyltetrazolium bromide (MTT) and quantified by absorbance at 595 nm. Percent viability was determined by comparison with vehicle-treated control samples. To measure apoptosis, DNA content (propidium iodide or sytox staining), externalized phosphatidylserine (Annexin V) and caspase activation (DEVDase activity) of the cells were determined using the CaspaTag *in situ* apoptosis detection kit (Millipore) and Dead Cell Apoptosis Kit with Annexin V APC and SYTOX® Green (Molecular probes). Labeled cells were analyzed using a FACS Calibur™ flow cytometer (BD Biosciences). Data were processed using FlowJo software (TreeStar).

### Western blot analysis and mitochondrial fractionation

Mitochondrial fractionation from cultured cells was performed with a Mitochondrial Isolation kit (Thermo Scientific) as described in the manufacturer’s instructions. For western blot analysis, proteins were separated on 8-12% SDS-polyacrylamide gels and transferred to polyvinyl difluoride membranes (Millipore). Primary antibodies were diluted 100–5,000-fold, and horseradish peroxidase-conjugated mouse or rabbit secondary antibodies (KLP Inc.) were diluted 5,000-fold. The ECL reagent (GE Healthcare) was used for chemiluminescence detection with a LAS 4000 imager (GE Healthcare).

### Tumor xenograft experiment

All experiments involving animals were approved by the Ulsan National Institute of Science and Technology Animal Care and Use Committee (approval number: UNISTIACUC-12-003-A). Cancer cells (7 × 10^6^ 22Rv1 or 1 × 10^7^ MDA-MB-231) were suspended in sterile 200 μl PBS. 22Rv1 cells were injected subcutaneously into both flanks of 8-week-old BALB/c nu/nu male mice (Japan SLC Inc.). MDA-MB-231 cells were orthotopically injected into the mammary fat pad of 8-week-old BALB/c nu/nu female mice. Gamitrinib or vehicle (DMSO) dissolved in 20% Cremophor EL (Sigma) in PBS was injected intraperitoneally (*i.p.*), and DOX diluted in PBS was injected intravenously (*i.v.*). The mice were treated with 10 mg/kg gamitrinib and/or 3 mg/kg DOX twice a week according to the group. Tumors were measured daily with a caliper and tumor volume was calculated using the formula V = 1/2 × (width)^2^ × length. At the end of the experiment the animals were euthanized, and organs including brain, heart, kidney, liver, lung, spleen, stomach, intestine, and testis, and tumors were collected for histologic or western blot analyses. Blood was also collected for measurement of serum creatine phosphokinase activity using the Indiko and Konelab System CK (Thermo Scientific) according to the manufacturer’s instructions.

### RNA extraction and reverse transcript-PCR

Total RNA was prepared from cells suspended in cold PBS using the RNeasy mini kit (QIAGEN), and cDNA was synthesized using the ProtoScript® First Strand cDNA Synthesis Kit (New England Biolabs) using an oligo(dT) primer. The PCR reaction was performed in a Mastercycler PCR machine (Eppendorf) with the following sets of oligonucleotide primers: NOXA, 5′-GTGCCCTTGGAAACGGAAGA-3′ and 5′-CCAGCCGCCCAGTCTAATCA-3′; PUMA, 5′-CAGACTGTGAATCCTGTGCT-3′ and 5′-ACAGTATCTTACAGGCTGGG-3′; DR5, 5′-TGCAGCCGTAGTCTTGATTG-3′ and 5′-GAGTCAAAGGGCACCAAGTC-3′; Bcl-2, 5′-TTTTAGGAGACCGAAGTCCG-3′ and 5′-AGCCAACGTGCCATGTGCTA-3′; Bim, 5′- ATGGCAAAGCAACCTTCTGA-3′ and 5′-GGAAGCCATTGCACTGAGA-3′; CHOP, 5′-CTTTCTCCTTCGGGACACTG-3′ and 5′-AGCCGTTCATTCTCTTCAGC-3′ GAPDH, 5′-GGGAAGCTTGTCATCAATG-3′ and 5′-GCAGTGATGGCATGGACT-3′.

### Statistical analyses

Data from MTT assay (triplicate experiments independently repeated at least two times) were averaged and statistically analyzed by unpaired *t*-test using Prism 5.0 (GraphPad). A *p*-value less than 0.05 was considered significant. To investigate the synergistic efficacy of the drug combination, the combination index (CI) was determined according to the Chou-Talalay method using CalcuSyn software version 2.1 (Biosoft) [23].

## Results

### Gamitrinib-doxorubicin combination treatment showed synergistic enhancement of cytotoxicity in various cancer cell lines

**Figure 1:**
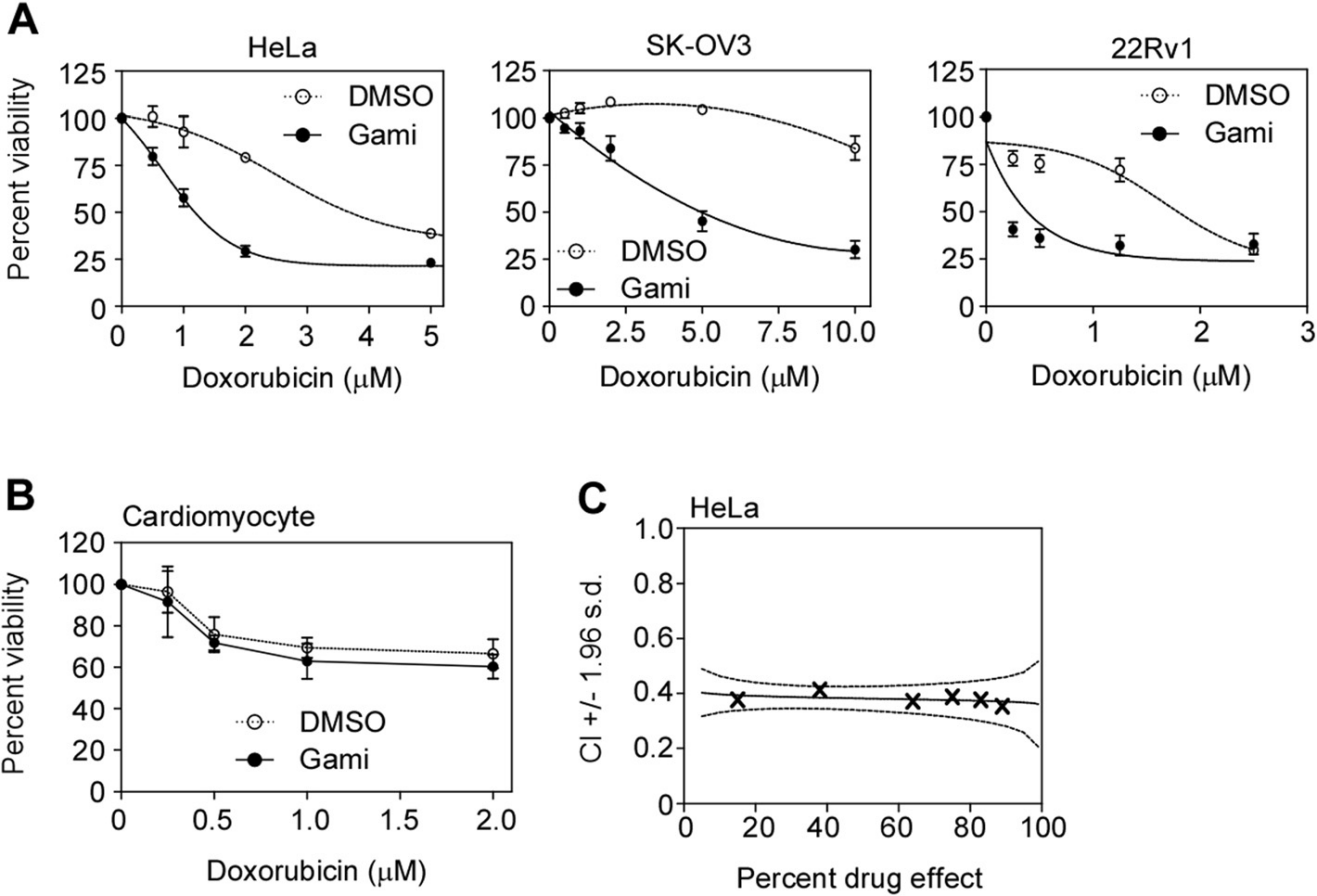
**Combination treatment with DOX and gamitrinib. (A)** Gamitrinib sensitizes cancer cells to DOX treatment. HeLa, SK-OV3, and 22Rv1 cells were treated with various concentrations of DOX in the absence (open circles) or presence of 5 μM, 10 μM, and 2.5 μM gamitrinib (closed circles), respectively, for 24 hours. Cell viability was analyzed by MTT assay. Percent viability was expressed as a percentage relative to 0 μM DOX-treatment. **(B)** Effect of combination treatment on cardiomyocytes. Mouse primary cardiomyocytes were treated in the absence (open circles) or presence (closed circles) of 5 μM gamitrinib for 24 hours and analyzed by MTT assay. **(C)** Graphical representation of combination index (CI) for HeLa cells. The MTT data were analyzed using CalcuSyn software to generate CI values. The 95% confidence interval of CI (mean ± 1.96 × standard deviation) is depicted as dotted lines.

To investigate the effect of combination treatment with DOX and gamitrinib, cancer cell lines originating from cervix (HeLa), ovary (SK-OV3), and prostate (22Rv1) were treated with the drugs as single agents or in combination. Gamitrinib sensitized HeLa, SK-OV3, and 22Rv1 cells to a wide range of DOX concentrations (Figure 1A) and the drug combination resulted in cancer cell death at suboptimal concentrations (Additional file 1: Figure S1A). In contrast to the effect on cancer cells, gamitrinib did not sensitize cardiomyocytes to DOX treatment (Figure 1B). Next, we calculated the combination index (CI) using the Chou-Talalay method [23]. The DOX and gamitrinib combination showed synergistic anticancer activity (CI < 0.9) in all cancer cell types tested at a 50% effective dose: high synergism (CI < 0.7) for HeLa (Figure 1C), A172 (glioblastoma), ACHN (renal cell carcinoma), SK-HEP-1 (hepatocellular carcinoma), NCI-H460 (lung carcinoma), and SK-OV-3; and moderate synergism (0.7 < CI < 0.9) for 22Rv1 and MDA-MB-231 cells (Table 1).

**Table 1:** **Combination Index (CI) values at ED**_**50 **_**and ED**_**75 **_**in various cancer cell lines**

**Cell line**	**Origin**	**Drug ratio**	**CI at ED**_**50**_	**CI at ED**_**75**_
		**DOX : Gami**		
HeLa	cervix	1 : 5	0.33 ± 0.03	0.41 ± 0.03
22Rv1	prostate	1 : 10	0.81 ± 0.18	0.53 ± 0.09
A172	brain	1 : 10	0.45 ± 0.28	0.76 ± 0.27
ACHN	kidney	1 : 2	0.34 ± 0.11	0.20 ± 0.07
SK-Hep1	liver	1 : 5	0.31 ± 0.05	0.29 ± 0.03
NCI-H460	lung	1 : 5	0.32 ± 0.19	0.45 ± 0.29
SK-OV3	ovary	1 : 1	0.58 ± 0.13	0.95 ± 0.28
MDA-MB-231	breast	5 : 1	0.73 ± 0.27	0.46 ± 0.24

NOTE: Cancer cells were treated with various concentrations of drugs at a fixed ratio as indicated. CI values at 50% (ED_50_) and 75% effective doses (ED_75_) were calculated from isobologram analysis. Data are the mean ± 1.96 s.d. (95% confidence interval) of two independent experiments performed in triplicate.

### Combination of DOX and gamitrinib augments apoptotic cell death

**Figure 2:**
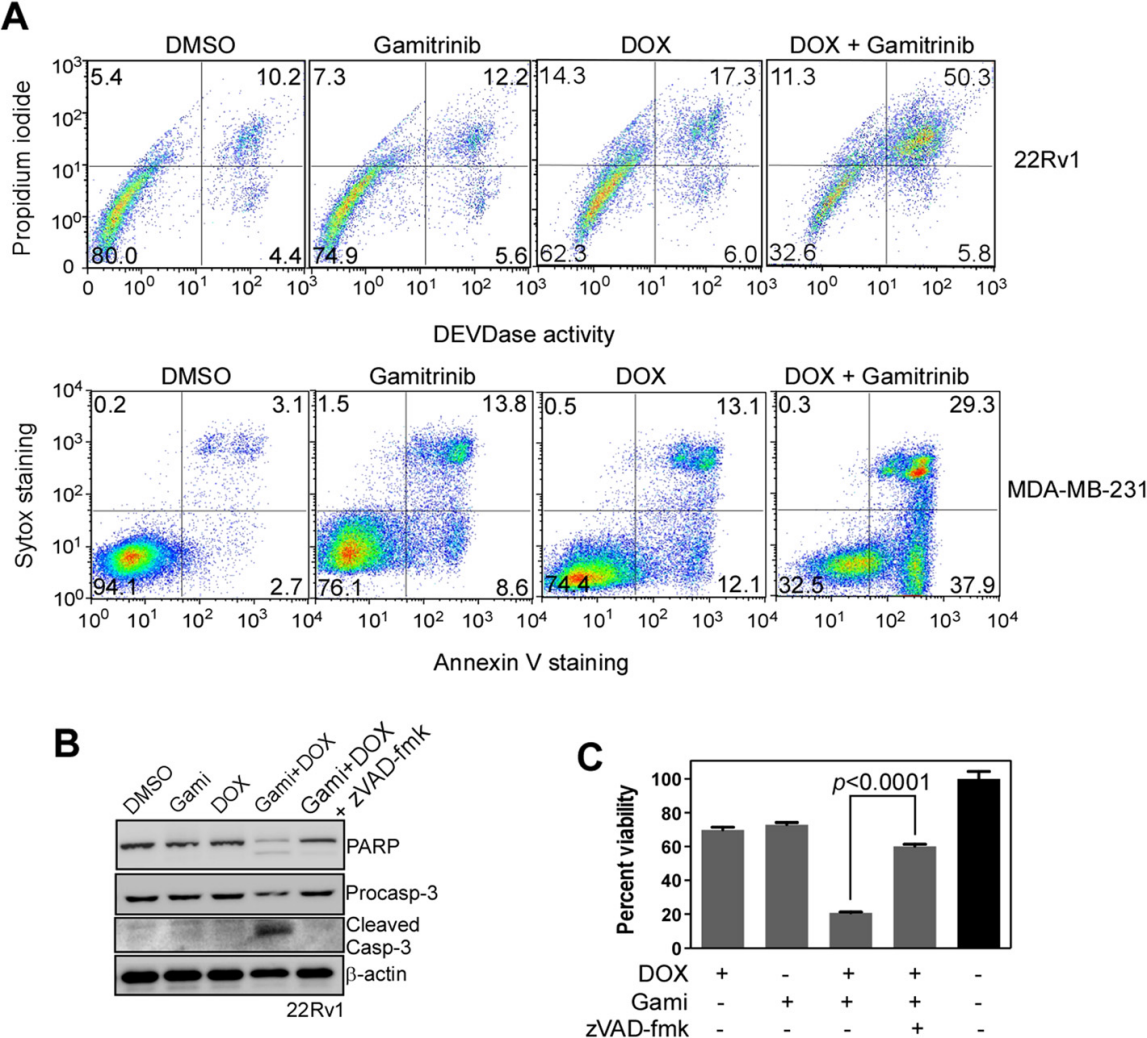
**Induction of apoptosis by combination treatment. (A)** Cell death analysis. 22Rv1 and MDA-MB-231 cells were treated with 0.25 μM DOX/2.5 μM gamitrinib and 10 μM DOX/5 μM gamitrinib, respectively, as single agents or combination treatment for 24 hours, and analyzed for propidium iodide, Sytox, Annexin V staining, and DEVDase activity by flow cytometry. The percentage of cells in each quadrant is indicated. **(B)** Caspase activation by the drug combination. 22Rv1 cells were treated with 0.25 μM DOX and 2.5 μM gamitrinib, and analyzed by western blotting. The pan-caspase inhibitor zVAD-fmk was used at a concentration of 10 μM. **(C)** Effect of caspase inhibitor on the drug combination treatment. 22Rv1 cells were treated with 0.25 μM DOX, 2.5 μM gamitrinib, and 10 μM zVAD-fmk as indicated and analyzed by MTT assay. Percent viability compared with DMSO-treated control is shown. Data are the mean ± SEM of duplicated three independent experiments.

Single drug treatment at a suboptimal concentration did not increase caspase activity significantly compared with the DMSO-treated control, whereas the drug combination dramatically increased caspase activity and concomitant cell death in 22Rv1 and MDA-MB-231 cells (Figure 2A). Consistently, cell extracts from 22Rv1 and SK-OV3 showed caspase activation for the combined drug treatment but not for single agent treatment (Figures 2B; Additional file 1: Figure S1B). Activation of caspase by the drug combination was completely inhibited by the pan-caspase inhibitor zVAD-fmk in 22Rv1 (Figure 2B). In addition, the enhanced cytotoxic activity of the drug combination was significantly abrogated by zVAD-fmk (Figures 2C; Additional file 1: Figure S1C). These data suggest that the drug combination activates caspases and triggers the apoptotic cell death program.

### Gamitrinib and DOX combination treatment activates expression of CHOP and Bim

**Figure 3:**
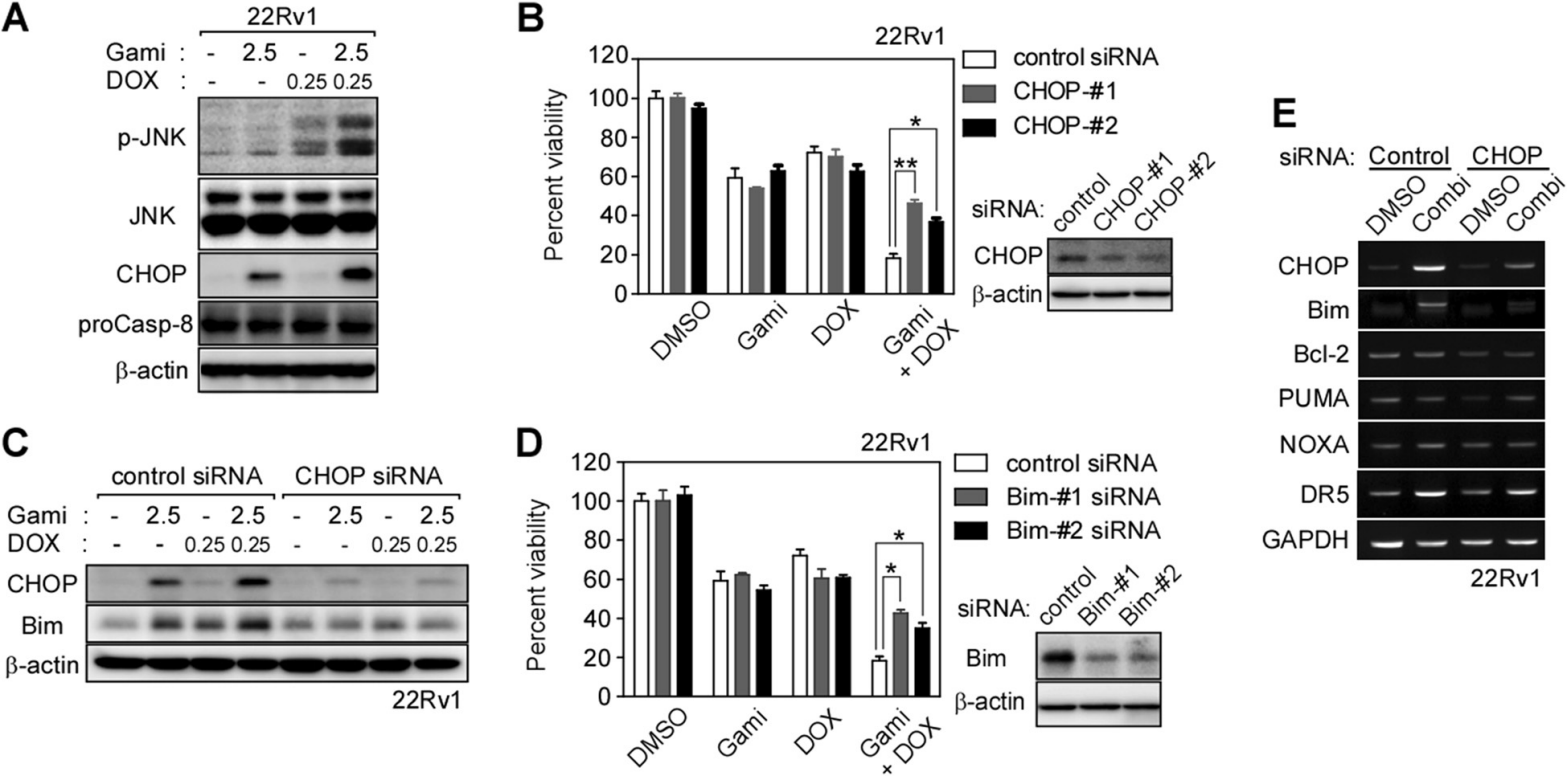
**Effect of drug combination on the expression of CHOP and Bim. (A)** CHOP induction and JNK phosphorylation. 22Rv1 cells were treated with DOX and gamitrinib alone or in combination as indicated and the whole cell lysate was analyzed by western blotting. **(B)** CHOP knockdown. 22Rv1 cells were treated with control or CHOP siRNAs for 24 hours and then with 0.25 μM DOX and 2.5 μM gamitrinib for 24 hours as indicated. The cell viability was analyzed by MTT assay. Data are mean ± SEM of two independent experiments performed in triplicate. *, *p* < 0.05; **, *p* < 0.004. **(C)** CHOP knockdown and Bim expression. After treatment with control or CHOP-#1 siRNAs, 22Rv1 cells were incubated with DOX and gamitrinib alone or in combination for 24 hours as indicated and cell extracts were analyzed by western blotting. **(D)** Effect of combination drug treatment after Bim silencing. After treatment with Bim siRNA, 22Rv1 cells were incubated with 0.25 μM DOX and 2.5 μM gamitrinib for 24 hours as indicated and cell viability was analyzed by MTT assay. Data are mean ± SEM of two independent experiments performed in triplicate. *, *p* < 0.05. **(E)** Expression of Bcl-2 family proteins and DR5. 22Rv1 cells were treated with 0.25 μM DOX and 2.5 μM gamitrinib for 24 hours as indicated. After extraction and reverse transcription of RNA, the cDNA of interest was amplified by PCR.

We investigated the effect of combined DOX and gamitrinib on stress signaling and found that c-Jun N-terminal kinase (JNK) activation (phosphorylation) and C/EBP homologous protein (CHOP) induction were increased more by the drug combination than by single agent treatment in 22Rv1 cells (Figure 3A). Treatment with CHOP-specific siRNA reduced the enhanced cytotoxicity after combined drug treatment but did not affect cytotoxicity of single agent treatment (Figure 3B), whereas JNK-specific siRNA did not affect the cytotoxic activity of the drugs singly or in combination (Additional file 1: Figure S2A). These data suggest that, in 22Rv1 cells, induction of CHOP is required for the combination effect of the drugs. As a downstream effector, the expression of Bim was enhanced by the drug combination in 22Rv1 cells and knockdown of CHOP compromised the up-regulation of Bim (Figure 3C). Another prostate cancer cell PC3 similarly showed synergistic induction of cell death and enhanced expression of CHOP and Bim by the drug combination (Additional file 1: Figure S2B). DOX alone slightly increased expression of Bim (Figure 3C; Additional file 1: S2B), which can occur through a CHOP-independent mechanism as previously reported [24,25]. Inactivation of Bim by siRNA treatment compromised the enhanced cytotoxicity of the drug combination (Figure 3D). Bim transcription was elevated, but expression of other Bcl-2 family proteins, such as Bcl-2, Puma, and Noxa, was not affected by the drug combination (Figure 3E). Expression of death receptor 5 (DR5) was elevated by the drug combination in a CHOP-dependent manner (Figure 3E) as described previously [26], while procaspase-8, recruited to the death inducing signaling complex (DISC) after DR5 activation [27], was not cleaved at all (Figure 3A). These data further suggest the crucial role of Bim expression in the drug combination.

### Gamitrinib and DOX combination treatment enhances mitochondrial localization of Bim and Bax

**Figure 4:**
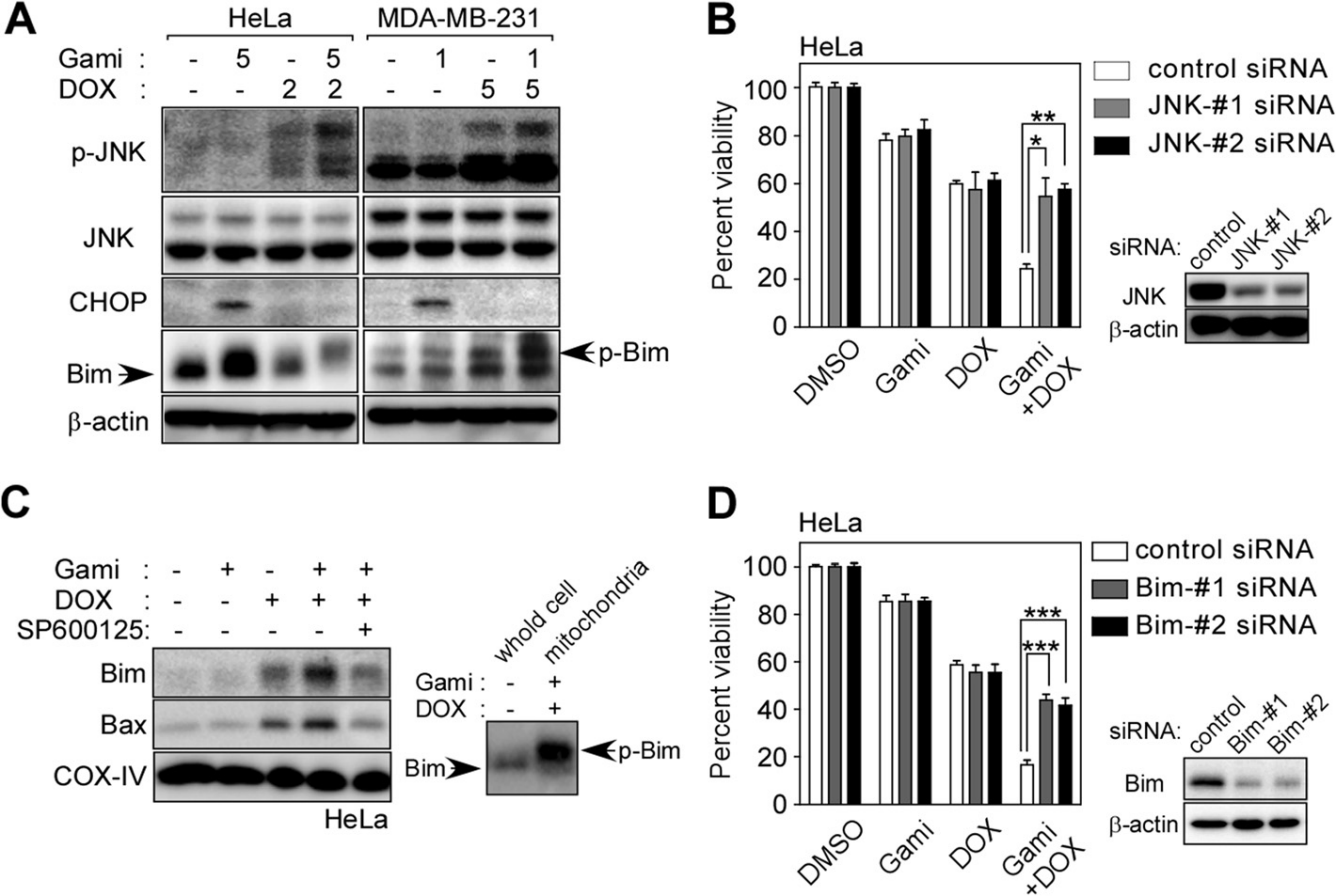
**Enhancement of JNK-mediated Bim phosphorylation by drug combination treatment. (A)** Phosphorylation of JNK and Bim. HeLa and MDA-MB-231 cells were treated with DOX and gamitrinib alone or in combination for 24 hours as indicated and analyzed by western blotting. **(B)** JNK silencing compromises the synergistic effect of combination treatment. HeLa cells were treated with JNK siRNA prior to treatment with 2 μM DOX and 5 μM gamitrinib as indicated for 24 hours. Cell viability was analyzed by MTT assay. Data are mean ± SEM of two independent experiments performed in triplicate. *, *p* < 0.02; **, *p* = 0.0004. **(C)** Mitochondrial accumulation of Bim and Bax. After treatment of HeLa cells with 5 μM gamitrinib, 2 μM DOX, or 10 μM SP600125 as indicated, mitochondria were fractionated and analyzed by western blotting (left). Bim phosphorylation in the whole cell extract and the mitochondrial fraction (right). **(D)** Effect of Bim silencing. After treatment with control or Bim-specific siRNAs, HeLa cells were incubated with 2 μM DOX and 5 μM gamitrinib for 24 hours as indicated and analyzed by MTT assay. Data show mean ± SEM of two independent experiments performed in triplicate. ***, *p* < 0.0001.

In contrast to 22Rv1 cells, the drug combination did not induce CHOP expression in HeLa and MDA-MB-231 cells (Figure 4A). In HeLa cells, knockdown of JNK compromised the enhanced cytotoxicity of the drug combination (Figure 4B) whereas knockdown of CHOP did not affect the drug synergism (Additional file 1: Figure S2C). These data suggest that there are context-dependent disparate stress responses to the drug combination, and the JNK pathway, but not CHOP, can be critically involved in the synergistic combination effect in certain cancer cell types. Previous studies have shown that JNK can activate Bim through phosphorylation to trigger Bax-dependent mitochondrial apoptosis [28,29]. Phosphorylated Bim was detected in HeLa and MDA-MB-231 cells, but not in 22Rv1 (Figures 3C and 4A). Bim protein in HeLa cells was reduced by the drug combination, but the proteasomal inhibitor MG132 largely elevated the Bim protein level (Additional file 1: Figure S3A). Similarly, CHOP depletion in the drug combination was also recovered by MG132 treatment (Additional file 1: Figure S3A). The data suggest that turnover rates of CHOP and phosphorylated Bim can be higher in certain cancer cell types as a result of proteasomal degradation [30,31]. Treatment with the drug combination caused increased accumulation of Bim and Bax in the mitochondria, which was significantly reduced by the JNK inhibitor SP600125 (Figure 4C; Additional file 1: Figure S3B) [32]. The Bim that accumulated in mitochondria was a slow-migrating phospho-form of the protein (Figure 4C, right panel). The combination effect was compromised by treatment with Bim siRNA (Figure 4D), further supporting the important role of the proapoptotic Bcl-2 protein in the drug-induced stress response [33].

### Drug combination treatment effectively inhibited tumor growth *in vivo* without aggravating cardiotoxic side effects

**Figure 5:**
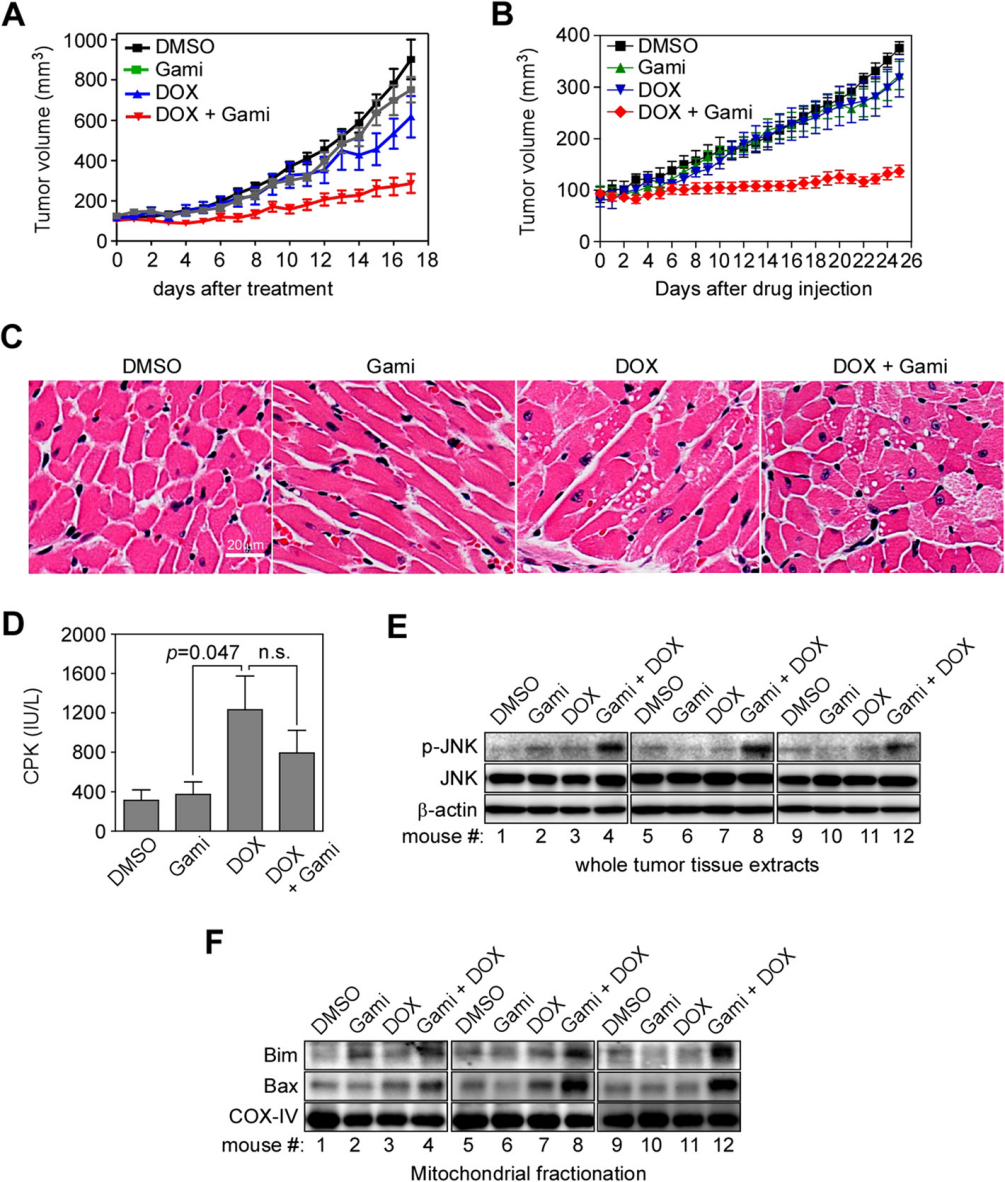
**Drug combination effect *in vivo. *(A)** Prostate cancer xenograft. 22Rv1 cells (7 × 10^6^ cells) were injected subcutaneously into both flanks of nude mouse (5 mice/group). After tumors were established, mice were treated twice a week with 3 mg/kg DOX *i.v.* and 10 mg/kg gamitrinib *i.p*. as single agents or in combination. **(B)** Orthotopic xenograft of breast cancer cells. MDA-MB-231 cells (1 × 10^7^ cells) were grown in mammary fat pads of nude mouse (3 mice/group). Mice were treated twice a week with 3 mg/kg DOX *i.v.* and 10 mg/Kg gamitrinib *i.p.* as single agents or in combination. **(C)** Hematoxylin and eosin staining of heart tissues. At the end of the experiment in **(B)**, heart ventricles were collected from the mice and analyzed by hematoxylin and eosin staining. Magnification, 200×. **(D)** Serum creatine phosphokinase activity. Blood was drawn from mice at the end of experiments in **(B)** and serum creatine phosphokinase (CPK) activity was measured. n.s., *p* > 0.05. **(E)** JNK phosphorylation in tumor tissue. Tumor samples collected from the xenograft mice in **(B)** were analyzed by western blotting. **(F)** Accumulation of Bim and Bax in mitochondria. Mitochondrial fractionations isolated from the tumors in **(B)** were analyzed by western blotting.

We examined the anticancer activity of gamitrinib in the presence of DOX using a prostate cancer xenograft model with the hormone-independent relapsed human prostate cancer cell line 22Rv1 [34]. Single treatment with gamitrinib or DOX resulted in a slight reduction in tumor volume, whereas combination treatment dramatically suppressed tumor growth (Figure 5A). Because DOX is frequently used to treat early and metastatic breast cancers in the clinic [35], we tested the effect of the drug combination on an orthotopic xenograft model with the triple negative (lack of ER, PR, and HER2 expression) metastatic breast cancer cell line MDA-MB-231 [36]. The tumor growth was strongly inhibited by the drug combination but not by single agent treatment (Figure 5B). Histologic analysis did not show any prominent differences among the groups (Additional file 1: Figure S4) except for the heart: a cardiotoxic phenotype of cytoplasmic vacuolization for DOX alone and in combination treatment (Figure 5C). The serum creatine phosphokinase (CPK) level was measured as an index of cardiotoxicity [37] at the end of the experiment (Figure 5D). DOX treatment alone increased the level of CPK, but there was no significant further increase in CPK levels by the drug combination (Figure 5D). These data suggest that the DOX-induced cardiotoxicity is not aggravated by the drug combination *in vivo*. The mechanism underlying the activity of the drug combination *in vivo* was consistent with the *in vitro* data: the drug combination synergistically increased the phosphorylation of JNK in whole tumor tissue extracts (Figure 5E) and the accumulation of Bim and Bax in mitochondrial fractions (Figure 5F).

## Discussion

In this study, DOX, one of the most widely used anticancer drugs, was combined with the mitochondria-stress inducer, gamitrinib, to exploit disparate stress pathways in cancer therapy. Combination of these agents synergistically increased cancer-specific cytotoxic activity through stimulation of JNK and CHOP stress signaling pathways and activation of the proapoptotic protein Bim. Importantly, the drug combination did not aggravate the well-known cardiotoxic side effects of DOX *in vitro* or *in vivo*.

Both gamitrinib [15,16] and DOX [38,39] have previously been shown to activate JNK and CHOP signaling pathways. Turning on these stress pathways activates the proapoptotic Bcl-2 family protein Bim through elevated gene expression and/or phosphorylation, leading to mitochondrial cell death [40,41]. As a result of simultaneous stimulatory effects on the stress pathways by DOX and gamitrinib, the drug combination is able to further increase the amount of Bim protein (through CHOP elevation) and/or mitochondrial accumulation of Bim (through JNK activation), leading to enhanced mitochondrial accumulation of Bax and synergistic induction of apoptotic cell death.

Combining cancer drugs with disparate mechanisms of action is a feasible strategy to increase therapeutic efficacy while avoiding unacceptable side effects of the drugs [42]. In this regard, combined treatment of DOX with other cancer drugs has been examined before and some of these combinations, for example with taxane or trastuzumab, have shown much more severe cardiotoxic side effects even at lower cumulative doses [18]. The combination of DOX and gamitrinib, however, did not aggravate cytotoxicity to cardiomyocytes *in vitro* or *in vivo*. We presume that cardiomyocytes are relatively resistant to gamitrinib because they are less dependent on mitochondrial chaperone functions to maintain protein homeostasis and cope with stresses under normal physiologic conditions.

## Conclusion

In conclusion, combined treatment of DOX and gamitrinib showed synergistically enhanced cancer-specific toxicity without aggravating cardiotoxic side effects. The drug combination can realize the full potential of the anticancer activity of the individual drugs and broaden the application of the drugs to various cancer types.

## Competing interest

The authors declare that they have no competing interest.

## Authors’ contributions

HKP and BHK participated in design of experiments, data analysis and interpretation, and manuscript preparation. HKP, JEL, JL, and DEJ participated in data collection. SAP and PGS provided technical and material support for animal experiments. All authors read and approved the final manuscript.

## Pre-publication history

The pre-publication history for this paper can be accessed here:

http://www.biomedcentral.com/1471-2407/14/431/prepub

## Supplementary Material

Additional file 1: Figure S1 Drug synergism and apoptosis induction. **Figure S2.** Drug combination effect and expression of CHOP, JNK, and Bim. **Figure S3.** Effect of MG132 and SP600125. **Figure S4.** Effects of drug treatment on normal tissues from xenografted mice. Click here for file
