# Supplementary material for: Combination treatment with doxorubicin and gamitrinib synergistically augments anticancer activity through enhanced activation of Bim
Source: BMC Cancer. 2014 Jun 13;14:431. doi: 10.1186/1471-2407-14-431 (PMC4072609; doi:10.1186/1471-2407-14-431)
Supplement: Additional file 1: Figure S1 — Drug synergism and apoptosis induction. Figure S2. Drug combination effect and expression of CHOP, JNK, and Bim. Figure S3. Effect of MG132 and SP600125. Figure S4. Effects of drug treatment on normal tissues from xenografted mice. [file 1471-2407-14-431-S1.pdf]

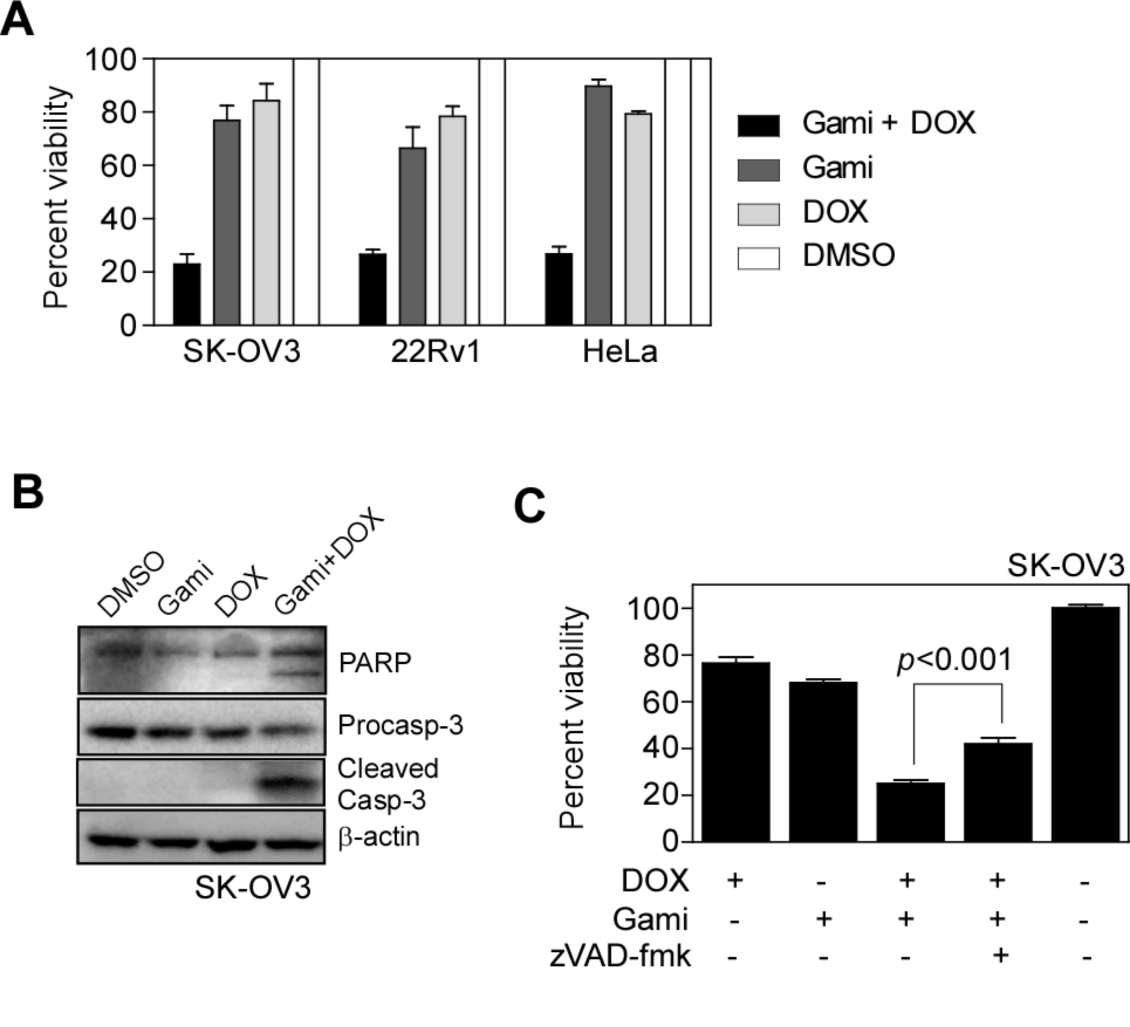

Supplementary Figure S1

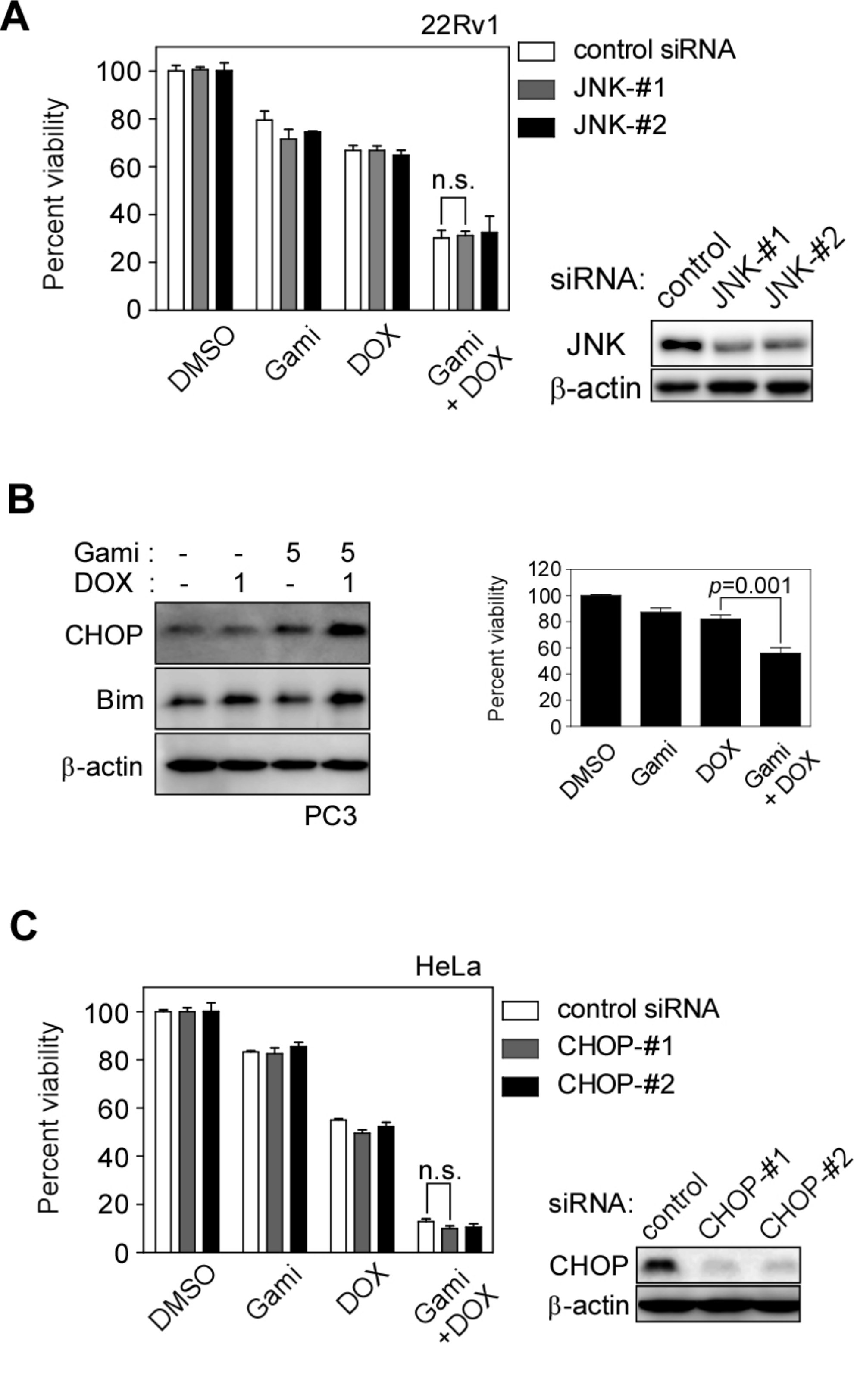

Supplementary Figure S2

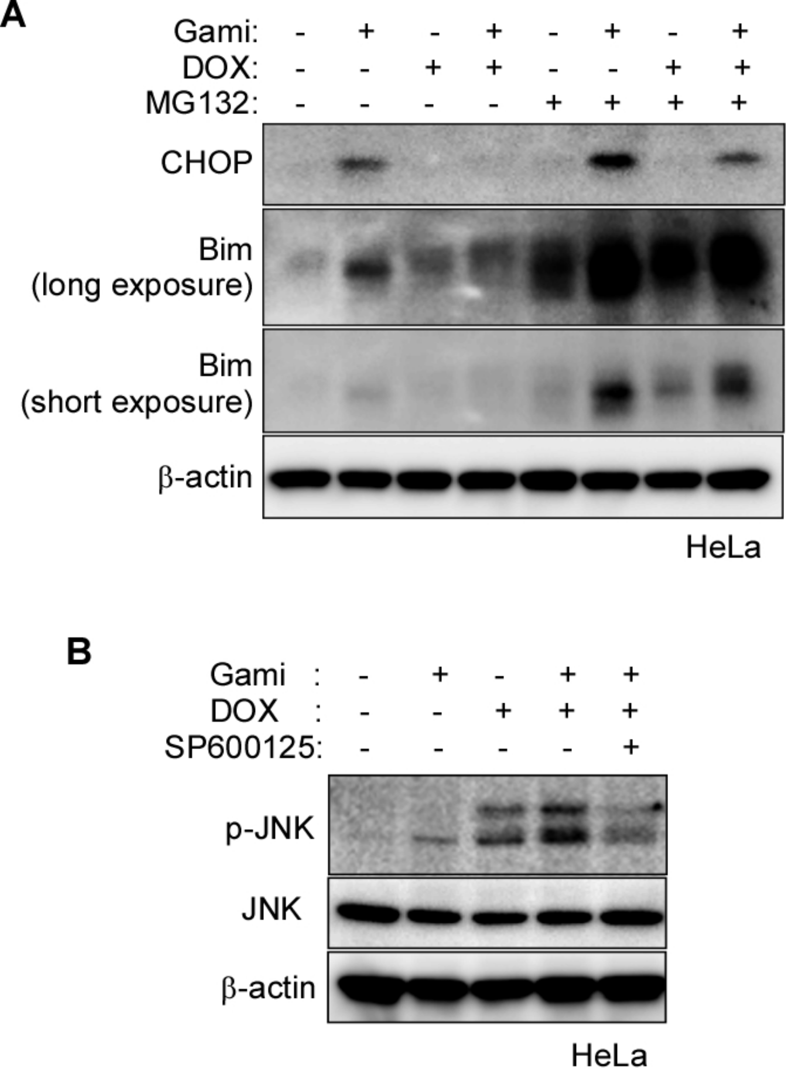

Supplementary Figure S3

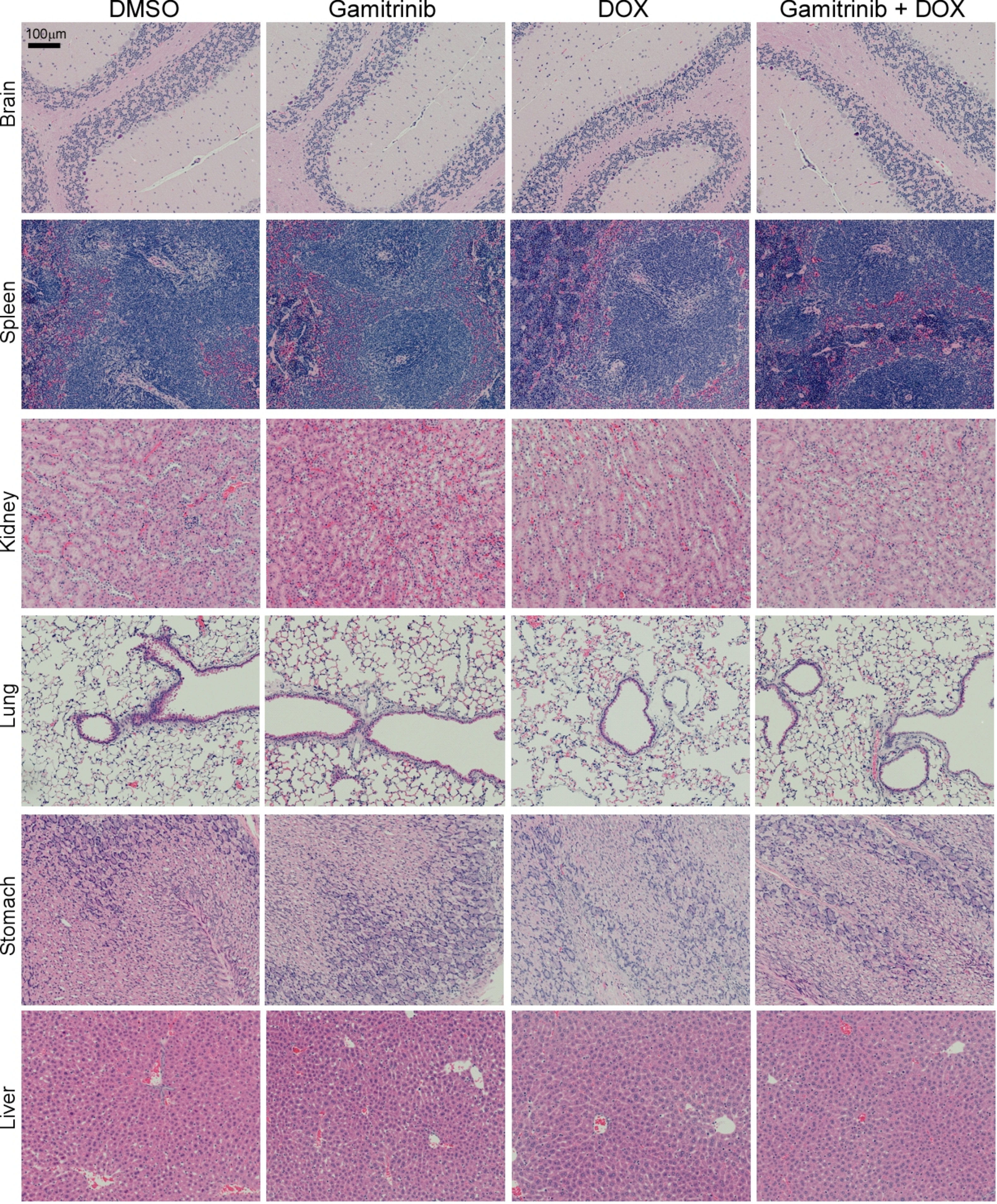

Supplementary Figure S4
